# Inflammation scores as prognostic biomarkers in small cell lung cancer: a systematic review and meta-analysis

**DOI:** 10.1186/s13643-021-01585-w

**Published:** 2021-01-28

**Authors:** Anne Winther-Larsen, Ninna Aggerholm-Pedersen, Birgitte Sandfeld-Paulsen

**Affiliations:** 1 Department of Clinical Biochemistry, Viborg Regional Hospital, Viborg, Denmark; 2 Department of Clinical Oncology, Aarhus University Hospital, Aarhus, Denmark; 3 Department of Clinical Biochemistry, Aarhus University Hospital, Palle Juul-Jensens Boulevard 99, 8200 Aarhus N, Denmark

**Keywords:** Small cell lung cancer, Inflammation scores, Neutrophil-to-lymphocyte ratio, Platelet-to-lymphocyte ratio, Glasgow prognostic score, Survival, Meta-analysis

## Abstract

**Background:**

Inflammation scores based on general inflammation markers as leucocyte count or C-reactive protein have been evaluated as prognostic markers of inferior survival in several cancers. In small cell lung cancer (SCLC), however, inflammation scores are less studied. In the present study, we set out to perform a systematic review and meta-analysis investigating reported associations between inflammation scores and overall survival (OS) in SCLC.

**Methods:**

A literature search was performed in PubMed, Embase, Scopus, and Web of Science following the Preferred Reporting Items for Systematic and Meta-Analyses (PRISMA) guidelines. Of the identified publications, only studies in English containing original data evaluating inflammation scores as a prognostic factor in SCLC patients were included. Hazard ratios (HRs) for OS were pooled in a random-effects model*.*

**Results:**

In total, 33 articles were included evaluating eight different inflammation scores in 7762 SCLC patients. Seven of the identified scores were based on leucocyte count. Neutrophil-to-lymphocyte ratio (NLR) and platelet-to-lymphocyte (PLR) ratio were the most frequently evaluated scores (NLR: *n* = 23; PLR: *n* = 22). For NLR, a meta-analysis including 16 studies demonstrated that patients with a high NLR had a significantly shorter OS compared to patients with a low NLR (pooled HR = 1.39 (95% CI, 1.23–1.56)). For PLR, an association with survival could not be confirmed in a meta-analysis performed based on eight studies (pooled HR = 1.20 (95% CI, 0.96–1.51)).

**Conclusions:**

This review identifies that inflammation scores based on general inflammation markers have some potential as prognostic biomarkers in SCLC. The meta-analyses indicated that NLR is associated with inferior OS, whereas an association between PLR and OS could not be confirmed. Thus, NLR could be a useful biomarker of OS in SCLC patients.

**Systematic review registration:**

The protocol for the study was submitted to the PROSPERO database (registration number CRD42020188553).

**Supplementary Information:**

The online version contains supplementary material available at 10.1186/s13643-021-01585-w.

## Background

Small cell lung cancer (SCLC) is the most aggressive and deadly form of lung cancer characterised by rapid growth, early metastasis, and high rates of acquired therapeutic resistance [1, 2]. Due to the nature of the disease, the majority of patients have metastatic disease at time of diagnosis leading to poor overall survival (OS) [3]. Over the last decades, improvements in cancer treatment have led to improved survival in non-small cell lung cancer (NSCLC) [4], but in SCLC patients, this impact on OS has been absent until lately, where the introduction of immunotherapy has shown promising results in clinical trials for this patient group [5, 6]. Though not all patients benefit from the available treatments, and for some patients, the course of the disease at time of diagnosis is fast and aggressive, therefore, the clinicians need guiding tools to predict the patient’s prognosis and the natural history of the disease. Moreover, to make improvements in the treatment of SCLC patients, we need prognostic markers that can identify patients who are at high risk of an inferior survival. By doing so, patients can be stratified into optimal treatment regimens or follow-up programmes which hopefully will lead to improved patient survival.

As one of the hallmarks of cancer [7], inflammation has been suggested as a prognostic marker [8]. Hence, general inflammation markers like C-reactive protein (CRP), leucocytes, or lymphocytes have been studied and shown some potential as prognostic markers in several cancers, even though results have been conflicting [9, 10]. Using individual inflammation markers as a measure of the inflammation status is a simplistic approach to a complex system. Therefore, inflammation scores that combine these general inflammation markers have been developed and proven to be prognostic markers of inferior survival in several cancers including NSCLC [10–15]. In SCLC, however, the prognostic value of inflammation scores is less studied, just as studies have shown inconsistent results [16, 17]. Therefore, we performed a systematic review to explore the literature on inflammation scores in SCLC. Furthermore, we performed a meta-analysis to investigate the prognostic value of pre-treatment neutrophil-to-lymphocyte ratio (NLR) and platelet-to-lymphocyte ratio (PLR) in SCLC patients.

## Materials and methods

### Data sources and search strings

A systematic search was carried out investigating the existing literature of inflammation scores in SCLC. The review was performed following the Preferred Reporting Items for Systematic Reviews and Meta-Analyses (PRISMA) guidelines [18]. The search was made in the databases PubMed, MEDLINE, Embase, and Web of Science on the 20th of March, 2020 with no time restriction. All databases were filtered for English, and PubMed and Embase were filtered for “not animals” in addition. Studies were selected using terms defining Lung cancer (“Lung cancer”, “Lung neoplasm*”, “Lung Neoplasms”[Mesh], “Lung carcinoma”), general inflammation markers (“Lymphocyte*”, “Lymphocytes”[Mesh], “Lymphocyte Count”[Mesh], “Neutrophil*”, “albumin*”, “Neutrophils”[Mesh], “CRP”, “C-reactive protein*”, “C-Reactive Protein”[Mesh], “albumin”, “Albumins”[Mesh]) and inflammation based scores (“glasgow prognostic score*”, “neutrophil to lymphocyte ratio*”, “neutrophil-lymphocyte ratio*”, “lymphocyte ratio*”, “inflammation score”, “inflammation-based score”, “inflammation index”). The full search string is available in Supplementary Text S1.

### Inclusion and exclusion criteria

The studies included in this review met the following inclusion criteria: (1) original data, (2) human studies, (3) patients with a pathologically proven histology of SCLC, and (4) studies evaluating a combination of general inflammation markers as a prognostic factor. Studies were excluded based on the following exclusion criteria: (1) language other than English; (2) papers without original data as reviews, meta-analyses, guidelines, editorials, comments, and letters to the editor; (3) conference abstracts or case reports including fewer than five cases; and (4) animal or in vitro studies. In case of two publications based entirely or partly on the same study population, the study containing the highest number of patients was included. According to the inclusion and exclusion criteria, two authors (BSP and AWL) screened the first 500 titles and abstracts to validate the inclusion and exclusion criteria. Disagreements were settled by discussion and consensus. The remaining titles and abstracts were screened by BSP. Two authors (BSP and AWL) read and included/excluded 30 randomly selected articles, and the remaining articles were assessed by BSP. The reference management tools Endnote (Clarivate Analytics) and Covidence (covidence.org) were used for identification of duplicates*.*

### Data extraction and quality assessment

Data extracted from the studies included name of first author, publication year, inclusion period, sample size, study design, and follow-up time. Furthermore, clinical characteristics of the study population and information on the inflammation score including cut-off, and risk estimates of the association with OS were extracted. Studies were split into two, and data extraction was performed by two authors (BSP and AWL); each author extracting data from half of the studies. All data extraction were checked by the other author. Both authors quality assessed the articles included in the study based on a modified version of the Quality of Prognosis Studies Tool (QUIPS) [19], and articles were rated as high quality, moderate, or low quality. The protocol for the study was submitted to the PROSPERO database (registration number CRD42020188553).

### Statistical analyses

For the individual inflammation score, a meta-analysis was performed if the score was evaluated in at least five studies with extractable risk estimates. Risk estimates included in the study were HR for the inflammation scores association with OS along with a 95% CI values or a beta coefficient and a standard error. Publication bias was evaluated by visual inspection of a funnel plot and by the Begg’s and Egger’s tests. Heterogeneity between the included studies was tested by using the Cochran *Q* and *I*^2^ [20], where *I*^2^ < 50% and *p* > 0.10 were set as cut-offs to define heterogeneity. In case of no significant heterogeneity, a fixed-effects model was applied; otherwise, a random-effects model was used. Sensitivity analyses were performed by excluding the low-quality studies and studies with predefined cut-offs to assess the robustness of the pooled estimate. Data were analysed by Stata software version 15.1 (Stata Corporation, College Station, TX, USA), and all *p* values were two-sided and considered significant if < 0.05.

## Results

### Study selection

**Fig. 1:**
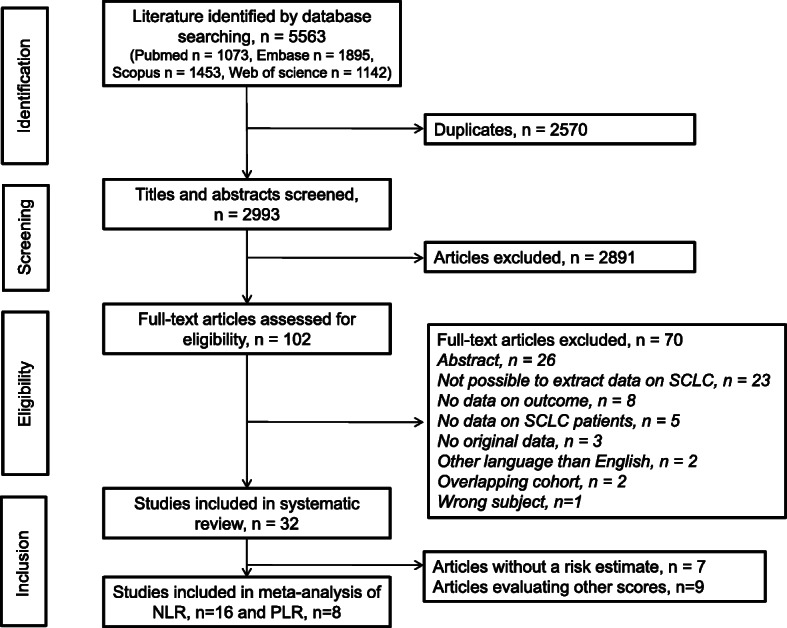
Flow chart of the inclusion and exclusion procedures. SCLC, small cell lung cancer

A total of 5563 publications were identified through searching the online databases; 2570 of these were excluded due to duplication. Titles and abstracts were screened for eligibility which led to exclusion of 2891 irrelevant articles. Full texts of the remaining 102 articles were thoroughly reviewed, and 70 articles were excluded due to various reasons: abstract, *n* = 26; not possible to extract data on SCLC, *n* = 23; no data on outcome, *n* = 8; no data on SCLC patients, *n* = 5; no original data, *n* = 3; other language than English, *n* = 2; overlapping cohort, *n* = 2; wrong subject, *n* = 1. Finally, 33 articles met the inclusion criteria for the current systematic review. The inclusion and exclusion procedures are illustrated in Fig. 1.

### Study description and quality assessment

Baseline characteristics of the studies included are listed in Table 1. In summary, all included studies were retrospective studies published between 2008 and 2020. The majority of studies were conducted in Asia (*n* = 23) [17, 21, 23–25, 27–30, 32, 33, 36–38, 41–44, 46–49], primarily China, whereas eight studies [16, 22, 26, 31, 34, 35, 50, 51] originated from Europe and two from the USA [39, 40, 45]. A total of 7762 patients with SCLC were included with the number of patients included in each study ranging from 46 to 938. In more than half of the studies (*n* = 19) [17, 21, 23–25, 27–30, 33, 36, 37, 41, 42, 44, 45, 48–50], the included patients were a mixture of patients with limited disease (LD) and patients with extended disease (ED), whereas only patients with LD were included in eight studies [16, 26, 31, 34, 40, 43, 46, 47] and only patients with ED were included in five studies [32, 35, 38, 39, 51]. For one study, the stage of disease was not described [22]. The presence of liver metastasis was described in seven studies [30, 32, 35, 38, 45, 48, 51] and ranged from 16 to 47% of patients with ED. The median/mean age of included patients varied from 56 to 72 years, and 6–50% of the included patients were female. Smoking was assessed in 26 studies [21, 23–27, 29–34, 36, 38–48, 50, 51] demonstrating that 28–100% of the patients had a history of smoking. Furthermore, the performance status was estimated in 26 studies [16, 17, 21–27, 29, 32–34, 36, 38–40, 42–45, 47–51] demonstrating that 33–100% of the patients were in a good performance defined by Eastern Cooperative Oncology Group Performance Status < 2 or Karnofsky performance scale ≥ 80.

**Table 1:** Studies investigating inflammation scores as prognostic biomarkers in small cell lung cancer patients (*n* = 33)

Author Year Country	Inclusion period	Study design ***N*** Age, median (range)	Clinical stage	Treatment	Female (%)	Current/ever smoker (%)	ECOG PS < 2 (%)	Inflammation score and cut-offs applied	Median follow-up, months (range)	Overall survival *U/M* HR (95% CI) or log-rank ***p*** value	Adjustment variables	Quality Score
Bernhardt 2008 Germany [16]	1999–2017	Retrospective *N* = 350 Median: 64 years (37–93)	LD: 350 (100)	Concurrent TCR: 350 (100)	137 (39)	NR	Median KPS: 80 (50–100)	NLR: 2.65 and 4.0	NR	U: 2.65: 0.86 (0.64–1.15) 4.0: 0.92 (0.71–1.19)		*Low quality*
Cao 2017 China [48]	January 2008–January 2010	Retrospective *N* = 707 Mean: 56 ± 10.15 years	LD: 419 (59) ED: 288 (41)	Platinum and etoposide: 707 (100) Thoracic RT: 294 (42)	253 (36)	442 (63)	KPS: > 80 point 543 (77) ≤ 80 points 164 (23)	NLR: 3.18^a^ PLR: 176.5^a^ LMR: 2.615^a^	562 (79%) events during follow-up	U: PLR not significant NLR p = 0.002 LMR p = 0.008 M: NLR: 1.030 (0.837–1.267) LMR: 1.053 (0.848-1.307)	Age, sex, KPS, smoking history, anaemia, lymphocyte count, NLR, PLR, LMR, LDH, ALP, surgery, thoracic irradiation, number of chemo cycles, number of metastatic sites, stage	*Moderate quality*
Deng 2017 China [21]	March 2007–December 2014	Retrospective *N* = 320 Median: 58 years (24–81)	LD: 122 (38) ED: 198 (62)	Surgery: 27 (8) Thoracic RT: 135 (42) PCI: 77 (24)	81 (25)	215 (63)	0: 104 (33) ≥ 1: 216 (67)	NLR: 2.65^a^ PLR: 125^a^	39.1 (3.2–85.4)	U: NLR < 0.001 PLR = 0.099 M: NLR: 1.35 (1.02–1.79)	NR	*Low quality*
Gioulbasanis^b^ 2012 Greece [22]	February 2006–March 2008	Retrospective *N* = 96 (SCLC *N* = 46) Median: 63 years (32–83)	NR	Platinum-based doublet CT: 96 (100)	12 (13)	NR	66 (69)	mGPS: 0/1/2	NR	U: SCLC: *p* = 0.008 M: no data for SCLC alone		*Low quality*
He 2015 China [23]	June 2006–December 2011	Retrospective *N* = 365 Median: 59 years (22–82)	LD: 201 (55) ED: 164 (45)	Etoposide-based CT: 191 (52) Irinotecan-based CT: 171 (47) Thoracic RT: 139 (38) PCI: 86 (24)	55 (15)	291 (80)	338 (93)	ALI: 19.5^a^	Last follow-up date September 2014	M: 1.617 (1.160 2.254)	Clinical stage, PS and LDH	*Moderate quality*
Hong 2015 [24] China	January 2000–December 2012	Retrospective *N* = 919 Median: 56 years (16–84)	LD: 552 (60) ED: 367 (40)	Surgery, RT, CT: 760 (83) No treatment: 159 (17)	284 (30)	567 (62)	760 (83)	NLR: 5 PLR: 250 SII: 1600	Last follow up data December 2014	U: NLR: *p* = 0.007 PLR: *p* = 0.004 SII: *p* < 0.001 M: NLR: 0.908 (0.721–1.144) PLR: 0.975 (0.783–1.215) SII: 1.377 (1.024–1.852)	Sex, age, smoking history, PS, stage, BMI, response to treatment, platelet count, HGB, MCV, MPV, PNI, LDH	*Low quality*
Kang 2014 Korea [25]	July 2006–October 2013	Retrospective *N* = 187 Median: 68 years (43–84)	LD: 67 (36) ED: 120 (64)	Platinum-based CT	25 (13)	172 (92)	163 (87)	NLR: 4^a^ PLR: 160^a^	40.28 (2.60–89.26)	U: NLR: *p* = 0.019 PLR: *p* = 0.467 M: NLR: 1.465 (1.012–2.119) PLR: 0.896 (0.628–1.280)	Stage and LDH	*Moderate quality*
Käsmann 2017 Germany [26]	2006–2014	Retrospective *N* = 65 ≤ 65 years: 36 (55%) > 65 years: 29 (45%)	LD: 65 (100)	Concurrent TCR: 35 (54) PCI: 47 (72)	25 (38)	18 (28)	47 (72)	NLR: 4 PLR: 180	NR	U: NLR: *p* = 0.001 PLR: non-significant M: NLR: 2.05 (1.06–3.95)	PS, pathologic lymph node, smoking, and PCI	*Low quality*
Kim 2016 Korea [27]	January 2010–October 2015	Retrospective *N* = 186 Mean: 69 years ± 9.4	LD: 64 (34) ED: 122 (66)	CT: 94 (50) TRC: 59 (32) Chest RT: 2 (1) Supportive care only 31 (17)	30 (16)	166 (89)	132 (71)	ALI: 31.1^a^	29.0 (19.7–38.3) 141 (83.5%) events during follow up	U: 2.10 (1.50–2.94) M: 1.67 (1.17–2.37)	NR	*Low quality*
Kim 2019 Korea [28]	2010–2016	Retrospective *N* = 157 Mean: 66 years ± 9.0	LD: 67 (43) ED: 90 (57)	NR	29 (19)	NR	NR	NLR: 2.48^a^ PLR: 110.43^a^	NR	U: Low NLR vs high NLR: 27.6 months vs 19.3 months, *p* = 0.151 Low PLR vs high PLR: 31.1 vs 19.3 months, p = 0.155		*Low quality*
Kurishima 2017 Japan [29]	April 1999–July 2016	Retrospective *N* = 319 Median: 71 years (49–94)	LD: 103 (32) ED: 216 (68)	CT: 276 (87) Supportive care: 43 (13)	46 (14)	304 (95)	192 (78) (PS 0–2)	mGPS: 0/1/2	NR	M: Score 1 vs 0: 1.23 (0.86–1.74) (*N* = 54) Score 2 vs 0: 2.04 C1.51–2.78) (*N* = 73)	NR	*Low quality*
Liu 2017 China [30]	January 2009–October 2013	Retrospective *N* = 139 Mean: 58 years ± 10.5	LD: 55 (39) ED: 83 (60) NR: 1 (1)	CT: 120 (86) RT: 61 (44)	32 (23)	100 (72)	NR	NLR:4.55^a^ PLR:148^a^	NR Follow up for at least 12 months	U: NLR: 3.309 (2.088–5.244) PLR: 1.813 (1.200–2.738) M: NLR: 2.093 (1.079–4.063) PLR: not significant, *p* = 0.332	Stage, metastatic disease, liver metastasis, adrenal metastasis, RT, CT, RBC, HGB, albumin, LDH	*Low quality*
Lohinai 2019 Hungary/Italy/Russia [31]	1999–2013, 3 centres	Retrospective *N* = 155 Median: 58 years (Range NR)	I: 60 II: 29 III: 40 Unknown: 26	Surgery, adjuvant CT: 100 (65)	41 (26)	141 (91)	NR	NLR: 2.258^a^ PLR: 111.253^a^	NR Last follow up date: April 2017	U: NLR: 1.621 (1.036–2.537) Low PLR vs. high PLR, median OS, 73.6 vs. 40.4 months, respectively, *p* = 0.084 M: NLR: 1.582 (1.010–2.478)	Surgery, pathologic lymph node, stage	*Moderate quality*
Minami 2017 Japan [32]	November 2007–June 2016	Retrospective *N* = 97 Mean: 71 years ± 8.7	IIIB: 18 (19) IV:79 (81)	Platinum-based CT: 97 (100) palliative RT: 4 (4)	20 (21)	67 (69)	66 (68)	mGPS: 0/1/2	NR 78 (80%) events during follow up	mGPS 0, 1 vs 2 U: 1.92 (1.19–3.07) M: 2.34 (1.27–4.31)	Brain metastasis, liver metastasis, bone metastasis, adrenal metastasis, PS, BMI, haemoglobin, creatinine clearence, sodium, LDH, ALP, CRP	*High quality*
Mirili 2018 Turkey	May 2007–February 2017	Retrospective *N* = 112 Median: 58 years (38–83)	LD: 26 (23) ED: 86 (77)	TCR: 35 (31) Platinum-based doublet CT: 62 (55) No treatment: 15 (13)	20 (18)	106 (95)	69 (62)	NLR: 3 / 4	8.4 (0.03–69.8) 89 (79.5%) events during follow-up	M: beta coef: 0.151 SE = 0.077	Whole body total lesion glycolysis, age, sex, stage	*Low quality*
Pan 2019 China [33]	January 2014–May 2016	Retrospective *N* = 73 Mean: 62 years (39–83)	LD: 29 (40) ED: 37 (51) Unknown: 7 (9)	Etoposide/carboplatin: 18 (25) etoposide/cisplatin: 40 (55) Other CT: 15 (21) local RT: 34 (47)	4 (6)	44/66 (67)	34/66 (52)	NLR: 3.8^a^	NR Last follow-up date: August 31, 2017 60 (82%) events during follow-up	U: high-NLR (*n* = 26) vs low-NLR groups (*n* = 34): 13.73 ± 1.87 vs. 13.22 ± 2.18 months; *P* = 0.785		*Low quality*
Sakin 2019 Turkey [34]	2012–2018	Retrospective. *N* = 113 Median: 61 years (35–83)	ED 113 (100)	Platinum/etoposide: 98 (87) Etoposide: 6 (1) Best supportive care: 9 (1)	21 (19)	113 (100)	72 (64)	NLR:3.0^a^ PLR: 150^a^ MLR:0.367^a^	6 (1–33) 92 (81%) events during follow-up	U: NLR: 2.23 (1.42–3.32) PLR: 1.69 (1.08–2.26) MLR: 0.61 (0.40–0.94) M: NLR: 2.26 (1.25–4.10) PLR and MLR: NR	NR	*Low quality*
Sakin 2019 Turkey [34]	1997–2017	Retrospective. *N* = 90 Median: 59 years (42–83)	LD: 90 (100)	Platinum/etoposide: 90 (100)	18 (20)	86 (96)	PS: 0–2: 77 (86)	NLR:3.0^a^ PLR: 150^a^ MLR:0.367^a^	NR 53 (59%) events during follow-up	U: NLR: 0.98 (0.89–1.08) PLR: 1.0 (0.99–1.00) MLR: 1.19 (0.41–3.43) M: NLR, PLR and MLR: NR		*Low quality*
Sedef 2018 [35] Turkey	September 2011–December 2017	Retrospective. *N* = 117 Median: 61 years (39–83)	ED: 117 (100)	Platinum/etoposide: 117 (100)	12 (10)	NR	NR	NLR: 3.28^a^ PLR: 139.8^a^	12 (range NR) 95 (81%) events during follow up	U: High NLR: 12 months vs low NLR 14 months *p* = 0.013 High PLR 13 months vs low PLR 13 months *p* = 0.66		*Low quality*
Shao 2015 China [36]	January 2000–March 2009	Retrospective. *N* = 112 Median: 62 years (45–82)	LD: 39 (35) ED: 73 (65)	Platinum/etoposide: 84 (75%) Platinum/irinotecan: 28 (25%)	14 (13)	105 (94)	103 (92)	NLR: 4.15^a^ PLR:150^a^	68.5 (range NR)	U: NLR: *p* = 0.001 PLR: *p* = 0.101 M: NLR: 1.56 (1.16–1.96) PLR: NR	Stage, PS	*Moderate quality*
Shen 2019 China [37]	September 2015–May 2019	Retrospective. *N* = 178 Mean: 61 ± 9.27	LD: 50 (28) ED: 128 (72)	Platinum/etoposide: 178 (100%)	36 (20)	NR	NR	HALP: 25.8^a^	NR	NR	NR	*Low quality*
Sonehara 2019 Japan [38]	January 2005–December 2018	Retrospective. *N* = 83. Median: 72 years (43–86)	ED: 83 (100)	Platinum/irinotecan: 33 (40%) Platinum/etoposide: 46 (55%) Etoposide 1 (1%) Best supportive care: 3 (4%)	13 (16)	79 (95)	60 (72)	mGPS: 0 / 1 / 2 PLR: 200	NR	U: mGPS 0 vs 1: 1.83 (1.06–3.15) mGPS 0 vs 2: 2.28 (1.21–4.30) PLR: 1.52 (0.94–2.47) M: mGPS 0 vs. 1: 1.31 (0.72–2.34) mGPS 0 vs. 2: 2.34 (1.16–4.73) PLR: 0.91 (0.35–1.56)	Age, PS, LDH, bone metastasis, lever metastasis	*Low quality*
Suzuki 2018 USA [39]	May 1998–September 2015	Retrospective *N* = 252 Median: 63 years (IQR 56–69)	ED: 252 (100)	Platinum: 240 (95) TRT ≥ 45Gy: 113 (45) PCI: 49 (19)	133 (53)	247 (98)	185 (73)	NLR: 4 PLR: 194.7	NR	U: NLR: 1.64 (1.27–2.11) PLR: 1.20 (0.93–1.55) M: NLR: 1.52 (1.17–1.98) PLR: non-significant, data not reported	Age, sex, PS, number of chemo cycles, TRT ≥ 45 Gy, PCI	*Moderate quality*
Suzuki 2019 Japan [40]	2002–2015	Retrospective. *N* = 122 Median: 65 years (Range NR)	LD: 122 (100)	Platinum/etoposide: 122 (100%)	61 (50)	111 (91)	118 (97)	NLR: 2.9^a^ PLR: 140.1^a^	NR	U: NLR: 1.68 (1.06–2.66) PLR: 1.85 (1.16–2.96) M: NLR: 1.86 (1.15–3.01) PLR: 1.72 (1.06–2.82)	Age, number of chemo cycles, stage	*Moderate quality*
Wang 2020 China [41]	January 2008–December 2009	Retrospective. *N* = 653 Median: 56 years (23–75)	LD: 384 (59) ED: 269 (41)	Platinum/etoposide: 653 (100%) RT: 267 (41%) Surgery: 22 (3%)	231 (35)	408 (63)	NR	SII:748.51^a^	NR	U: Low SII were associated with a prolonged OS 17 vs 12 months p < 0.001 M: 1.55 (1.30–1.86)	Age, sex, smoking, stage, LDH, distant metastasis, CRT, surgery + adjuvant CT	*Low quality*
Wang 2019 China [42]	March 2009–August 2015	Retrospective. *N* = 228 Median: 58 (39–71)	LD: 114(50) ED: 114 (50)	TRC platinum/etoposide 228 (100%)	69 (30)	181 (79)	KPS ≥ 80 186 (82)	SII: 479^a^ NLR: 2.3^a^ PLR: 125^a^ LMR: 6.08^a^	46 (range NR)	U: SII: 4.80 (3.42–6.74) PLR: 3.17 (2.15–4.66) NLR: 2.53 (1.84–3.48) LMR: 1.96 (1.40–2.75) M: SII: 2.67 (1.67–4.28) PLR: 1.95 (1.26–3.00) NLR: 1.38 (0.93–2.05) LMR: 1.32 (0.92–1.89)	Initial therapeutic response, extrapulmonary lesion, stage	*Low quality*
Wang 2014 China [17]	January 2005–December 2010	Retrospective. *N* = 114 Mean: 59 years	LD: 68 (60) ED: 46 (40)	Platinum/etoposide: 92 (80%)	25 (22)	NR	45 (39)	NLR: 3.0 PLR: 150	NR	U: NLR: chi2 = 5.641, *P* = 0.018 PLR: NR M: NLR: 1.70 (1.05–2.75) PLR: NR	PS, stage	*Moderate quality*
Wen 2017 China [43]	January 2005–December 2010	Retrospective. *N* = 452 Median: 56 years (27–82)	LD: 452 (100)	Platinum/etoposide: 376 (83%) Platinum/irinotecan: 76 (17%)	112 (25)	322 (71)	357 (79)	NLR: 4.0 PLR: NR	35 (range NR) NR	U: NLR: *p* = 0.004 PLR: *p* = 0.016 M: NLR: 2.04 (1.02–4.10) PLR: NR	Number of cycles, treatment modality, LDH, NSE, platinum status, response to treatment	*Moderate quality*
Wu 2020 [44] China	January 2008–October 2018	Retrospective. *N* = 146 Mean: 57 years (19–74)	LD: 59 (40) ED: 87 (60)	Platinum/etoposide: 146 (100%)	32 (22)	108 (74)	146 (100)	NLR: 3.0 PLR: 165	14 (5–138) 140 events during follow-up	U: NLR: 1.57 (1.08–2.29) PLR: 1.46 (1.02–2.11)		*Moderate quality*
Xie 2015 USA [45]	January 1997–December 2012	Retrospective. *N* = 938 Median: 68 years (27–91)	LD: 383 (41) ED: 555 (59)	CT: 777 (83%)	438 (47)	921 (99)	730 (78)	NLR: 5.0 PLR: 210^a^	10.8 (range NR) 856 (91%)events during follow-up	U: PLR *p* < 0.0001 NLR *p* < 0.0001 RDW *p* < 0.0001 M: LD PLR: 1.60 (1.18–2.18) Log_e_ (NLR): 1.16 (0.96–1.42) Log_e_ (RDW): 0.84 (0.24–2.94) M: ED Log_e_ (RDW): 2.81 (1.32–6.01) Log_e_ (NLR): 1.41 (1.24–1.59) PLR: 0.83 (0.67–1.02)	Age, sex, PS, chest irradiation, CT, liver metastasis, number of metastatic sites	*Low quality*
Zhang 2019 China [46]	January 2012–September 2015	Retrospective. *N* = 286 < 65 years: 220 (77%)	LD: I: 20 (7) II: 48 (17) III: 218 (76)	Platinum/etoposide: 236 (83%) Other not specified: 50 (17%)	84 (29)	161 (56)	NR	PLR: 152.1^a^	40 (4–74) 221 (77%) events during follow-up	U: *p* = 0.002 M: 1.33 (1.01–1.74)	Age, stage, treatment, initial treatment regimen, PCI	*Low quality*
Zheng 2018 China [47]	January 2010–December 2016	Retrospective. *N* = 153 Median: 59 years (23–80)	LD: I: 4 (3) II: 13 (8) III: 136 (89)	Platinum/etoposide: 153 (100%)	49 (32)	84 (55)	139 (90)	NLR: 2.55^a^ PLR: 125.7^a^	42.5 (5.8–93.2) 88 (52%) events during follow-up	U: NR M: NLR: 2.14 (95%CI NR) PLR: NR	NR	*Low quality*
Zhou 2014 China	January 2009–December 2011	Retrospective. *N* = 359 Median: 60 years (22–82)	LD: 163 (45) ED: 196 (55)	Platinum / irinotecan: 174 (47%) Platinum / etoposide: 185 (53%)	55 (15)	NR	341 (95)	mGPS: 0 / 1 / 2	NR 180 (50.1%) events during follow-up	U: 0 v 1 v 2: *p* < 0.001 M: 0 v 1 (*n* = 238v 110): 1.52 (1.08–2.13) 0 v 2 (*N* = 238 v 11): 5.23 (2.63–11.58)	PS, sex, stage	*High quality*

*ALI*, advanced lung cancer inflammation index (body mass index × albumin/NLR ); *ALP*, alkaline phosphate; *CT*, chemotherapy; *ECOG PS*, Eastern Cooperative Oncology Group performance status; *HALP*, haemoglobin × albumin × lymphocytes/platelet count; *HGB*, haemoglobin; *KPS*, Karnofsky performance scale; *LDH*, lactate dehydrogenase; *LMR*, lymphocyte-to-monocyte ratio; *M*, multivariate; *MCV*, mean cell volume; *MLR*, monocyte-to-lymphocyte ratio; *mGPS*, modified Glasgow Prognostic Score (CRP ≤ 10 mg/L and albumin ≥ 35 g/L: score 0; CRP > 10 mg/L and ≥ 35 g/L: score 1; CRP < 10 mg/L and ≤ 35 g/L: score 1; CRP > 10 mg/L and albumin < 35 g/L: score 2); *MPV*, mean platelet volume; *PCI*, prophylactic cranial irradiation; *PLR*, platelet-to-lymphocyte ratio; *PNI*, prognostic nutritional index; *RBC*, red blod cell count; *RT*, radiotherapy; *SII*, systemic immune-inflammation index (= platelet count × neutrophil count/lymphocyte count); *TCR*, thoracic chemo-radiotherapy; *U*, univariate

^a^Data-dependent cut point

^b^In the original paper, the score is named Glasgow prognostic score. However, the score is calculated as the mGPS and therefore evaluated as mGPS in this study

Eight different inflammation-based scores were identified. The inflammation-based scores neutrocyte-to-lymphocyte ratio (NLR) and platelet-to-lymphocyte ratio (PLR) were the most frequently evaluated (NLR: *n* = 23; PLR: *n* = 22). Other identified inflammation-based scores were modified Glasgow prognostic score (mGPS, *n* = 5), lymphocyte-to-monocyte ratio (LMR, *n* = 2), monocyte-to-lymphocyte ratio (MLR, *n* = 2), advanced lung cancer inflammation index (ALI, *n* = 2), systemic immune-inflammation index (SII, *n* = 2), and haemoglobin, albumin, lymphocyte, and platelet score (HALP, *n* = 1).

For mGPS, cut-offs were identical and predefined in all studies. For the remaining scores, predefined cut-offs were defined in seven studies [16, 17, 24, 26, 43, 44, 50], while data-dependent cut-offs were employed in 19 studies [21, 23, 25, 27, 28, 30, 31, 33–37, 40–42, 46–48, 51]. In two studies evaluating different scores, NLR was evaluated based on a predefined cut-off, whereas the cut-off for PLR were data dependent [39, 45]. As a consequence, cut-offs varied substantially for NLR, 2.3–5.0; PLR, 110.43–250; LMR, 2.615–6.08; ALI, 19.5–31.1; and SII, 748.51–1600. The inflammation scores were evaluated as prognostic biomarkers based on a blood sample collected at diagnosis of the lung cancer (*n* = 8) [21, 24–27, 29, 33, 50] or before start of treatment (*n* = 23) [16, 17, 22, 23, 28, 30–32, 34–37, 39–47, 49, 51]. For two studies [38, 48], the time of blood sampling was not reported. Based on the quality assessment, included studies were ranked from low to high quality; 21 studies were ranked low quality [16, 21, 22, 24, 26–30, 33–35, 37, 38, 41, 42, 45–47, 50, 51], ten studies ranked moderate quality [17, 23, 25, 31, 36, 39, 40, 43, 44, 48], and two studies ranked high quality [32, 49].

### Inflammation scores and overall survival

In the identified 33 studies, an adjusted risk estimate of mortality risk between patients with a low versus a high score could only be retrieved in 25 studies (NLR, 16 [17, 21, 24–26, 30, 31, 36, 39, 40, 42, 43, 45, 48, 50, 51]; PLR, 7 [24, 25, 38, 40, 42, 45, 46]; LMR, 2 [42, 48]; MLR, 2 [34, 51]; mGPS, 4 [29, 32, 38, 49]; SII, 3 [24, 41, 42]; ALI, 1 [23, 27]). Rating and adjustment variables for the individual study are listed in Table 1. The studies were rated from low quality to high quality. The applied adjustment variables were reported in 20 studies [17, 23–26, 30–32, 36, 38–43, 45, 46, 48–50] while no information on adjustment variables were reported in five studies [21, 27, 29, 47, 51]. For inflammation-based scores evaluated in more than five studies, a meta-analysis was performed.

#### NLR and overall survival

**Fig. 2:**
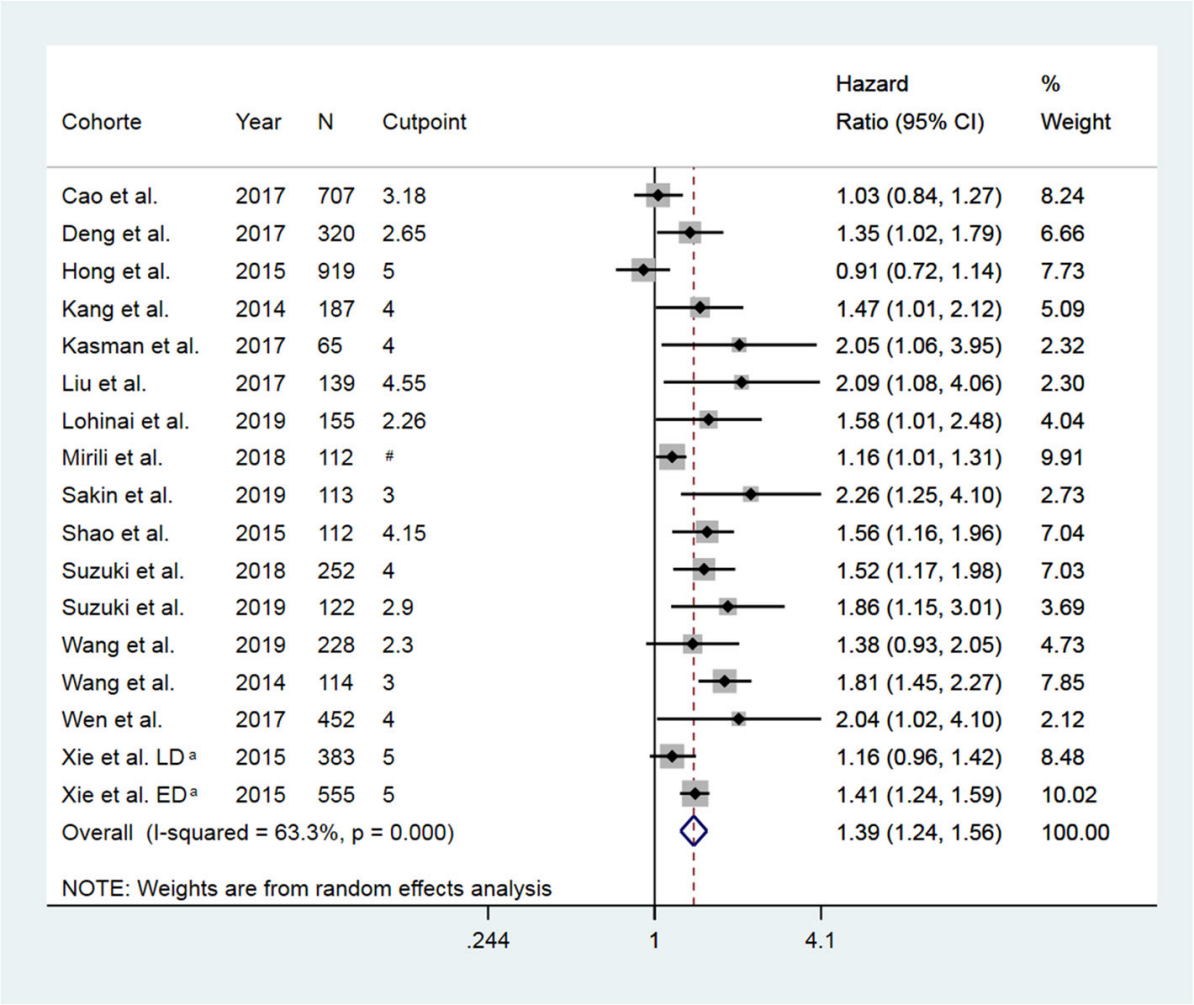
Forrest plot of hazard ratio for the association between neutrophil-to-lymphocyte ratio and overall survival in patients with small cell lung cancer. N, number of included patients; CI, confidence interval; LD, limited disease; ED, extended disease. ^#^ Two cut-points (NLR 3 and 4) were evaluated. ^a^NLR were evaluated in two individual subgroups: LD and ED

A substantial between-study heterogeneity was observed in the 16 studies evaluating NLR (*Q* = 43.62 on 16 df; *P* < 0.0001; *I*^2^ = 63.3%; *p* = 0.03), why a pooled HR was estimated using a random-effects model. A high NLR was found to be associated with a 39% increased risk of death in patients with SCLC (HR = 1.39 (95% CI, 1.23–1.56), Fig. 2).

Due to an observed asymmetry in the funnel plot, publication bias was suspected (Supplementary Figure 1), which was supported by the Eggers test (*p* = 0.04) and a tendency observed in the Begg’s test (*p* = 0.06). Hence, a Trim and Fill analysis was performed to account for absent studies, and an adjusted pooled random-effects HR was calculated. The pooled HR remained significant, even though the estimate was slightly reduced (HR = 1.23 (95% CI, 1.13–1.42)).

Since the meta-analysis included a large number of studies ranked low quality, a sensitivity analysis was performed including only the moderate quality ranked studies. Evaluating only the nine studies of moderate quality, we found a pooled HR of 1.51 (95% CI, 1.29–1.81). Furthermore, in nine studies, data-dependent cut-points were applied; hence, a sensitivity analysis was performed including only studies with predefined cut-points. Here we found a pooled HR of 1.34 (95% CI, 1.15–1.58).

#### PLR and overall survival

**Fig. 3:**
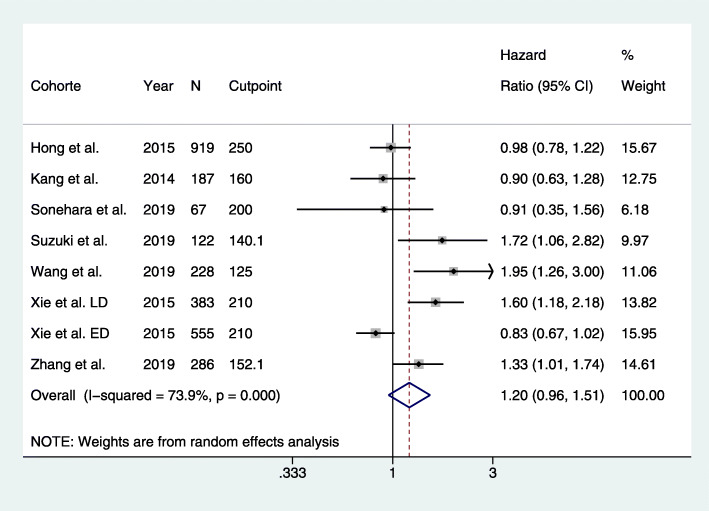
Forrest plot of hazard ratio for the association between platelet-to-lymphocyte ratio and overall survival in patients with small cell lung cancer. N, number of included patients; CI, confidence interval; LD, limited disease; ED, extended disease. ^a^PLR were evaluated in two individual subgroups: LD and ED

In the studies of PLR, a considerable between-study heterogeneity was also detected (*Q* = 26.87 on 7 df; *P* < 0.0001; *I*^2^ = 74%; *p* = 0.12), and again, a pooled HR was estimated using a random-effects model. A high PLR was associated with a 20% increased risk of death in patients with SCLC; however, the estimate of the risk increase was ranged from − 4 to 51% (HR = 1.20 (95% CI, 0.96–1.51), Fig. 3). An asymmetry in the funnel plot was observed and publication bias was suspected (Supplementary Figure 2). However, Begg’s test (*p* = 0.17) and Egger’s test (*p* = 0.22) did not identify publication bias.

## Discussion

In this systematic review, we explored the literature on inflammation scores as prognostic biomarkers in SCLC. We identified eight different inflammation scores and evaluated their ability to predict OS in SCLC. By the use of meta-analyses, we demonstrated that NLR leads to a 39% increase in mortality in SCLC patients. This finding was confirmed after taking the risk of publication bias into account, even though the pooled HR was reduced. Opposite, no association with OS could not be confirmed for PLR. All studies included in the review were retrospective studies, just as the overall quality of the studies was low. Nevertheless, the association was confirmed after omitting low-quality studies from the analysis. Regardless of these hesitations, as the first, this review collects the available literature on inflammation scores in SCLC and evaluates their potential as prognostic biomarkers in SCLC.

Inflammation is recognised as one of the hallmarks of cancer, why inflammation scores based on general inflammation markers have been demonstrated as a prognostic biomarker in several cancers [10–15]. Though, so far a hypothesis explaining the biological mechanisms behind these various inflammation scores, and especially, a hypothesis explaining why inflammation scores are associated with mortality, has been absent. A potential part of this puzzle could be interleukin-1β, as inhibition of interleukin-1β was shown to lead to a decrease in lung cancer incidence as well as mortality in atherosclerotic patients [52], and high levels of interleukin-1β has been observed along with anaemia, neutrophilia, lymphopenia, low levels of albumin, and increased CRP in patients with rheumatic disease [53]. In this comprehensive review, all identified inflammation scores, but mGPS, were based on lymphocyte count in various combinations with neutrophils, platelets, monocytes, albumin, haemoglobin, and BMI. NLR was the most frequently evaluated score (*n* = 23) closely followed by PLR (*n* = 22). For NLR, which can be a reflection of interleukin-1β associated neutrophilia and lymphopenia, high scores were related to inferior survival. Similarly, in the NLR-modified scores (ALI and SII) a high score was associated with inferior survival. For PLR, however, the association with OS could not be confirmed. A possible explanation for this could be that the PLR only includes one general inflammation marker (lymphocyte) affected by the interleukin-1β. Consequently, the HALP score, which is a modification of the PLR by including haemoglobin and albumin, should have an improved association with survival theoretically. Unfortunately, however, survival data were not reported for the HALP score [37]. The only score not including lymphocyte count is the mGPS, which is based on two other markers potentially reflecting interleukin-1β: albumin and CRP. We identified five studies [22, 29, 32, 38, 49] evaluating the prognostic potential of mGPS in a total of 954 SCLC patients with both LD and ED. A high mGPS score was overall associated with reduced OS, though one of the studies [22] did not report an adjusted HR. Due to the low number of studies, a meta-analysis could not be performed. However, in other cancer types like NSCLC, the prognostic value of mGPS has been established even in meta-analyses [54, 55] indicating that mGPS could be a valuable biomarker in SCLC as well.

In all scores except mGPS, data-dependent cut-offs based on survival were frequently applied (*n* = 19). When survival is used to decide how to define a cut-off in a given biomarker, the likelihood of the given biomarkers’ ability to be able to predict survival is enlarged enormously. Thus, this could be a potential bias to this study. Therefore, we performed a subgroup analysis excluding studies with data-dependent cut-offs and demonstrated only a slight reduction of the combined risk estimate indicating that data-dependent cut-offs were not a major bias in our study. Furthermore, due to the data-dependent nature of cut-offs, the cut-offs varied tremendously for all scores with a tendency of higher predefined cut-offs compared to data-dependent cut-offs.

Until now, only one systematic review on NLR in SCLC has been performed [56] identifying an association between NLR and OS. In the previous review, 21 studies were included of which six were posters from international conferences and two of these could not be identified in the PubMed, Embase, WOS, or Scopus databases. Moreover, a quality assessment was not performed in the previous review. Besides, in this study, 13 additional studies evaluating NLR as a prognostic marker in SCLC were identified owed to the comprehensive search, leading to a more wide-ranging evaluation of NLR as a prognostic biomarker.

The strength of this study is the comprehensive systematic review of the available literature on inflammation scores in SCLC. We used four internationally recognised databases applying broad search terms to include all inflammation scores available. Though we included specific search terms for well-known scores as NLR, PLR, and mGPS, thus, the likelihood of retrieving more results on these specific scores is present. To counter this potential skewness in identified scores, we included broad search terms in our search strategy including individual inflammation markers and various terms covering the inflammation score term. An additional strength of the review is the quality assessment performed by two authors. The quality assessment is essential to identify biases of a magnitude to affect study results. We applied a modified version of the QUIPS [19], which assesses six important domains being: study participation, study attrition, prognostic factor measurement, confounding, measurement and account, outcome measurement, and analysis and reporting.

Nevertheless, the study faces some limitations. Firstly, all studies were retrospective and the overall quality of the included studies was low. Additionally, substantial heterogeneity was observed between the included studies as *I*^2^ values of 63% for NLR and 74% for SCLC were observed. The *I*^2^ measure the variation in the estimates caused by-study differences. In general, an *I*^2^ > =50% indicate moderate heterogeneity and an *I*^2^ > 75% indicate substantial to considerable heterogeneity [57]. These thresholds are arbitrary. However, the *I*^2^ cannot stand alone but should be considered in combination with the forest plot. If the estimates vary but point towards the same conclusion in the forest plot as is seen for NLR (Fig. 1) and PLR (Fig. 2), a substantial heterogeneity can be present, but it would be of questionable clinical importance [57]. Finally, a language bias cannot be excluded as we only included studies written in English.

## Conclusion

This review identifies that inflammation scores based on general inflammation markers do have some potential as prognostic markers in SCLC patients. The conducted meta-analyses demonstrated that NLR was associated with mortality. Furthermore, mGPS showed some potential as a prognostic marker of inferior survival, though only in a limited number of studies. Hence, inflammation scores as NLR could be clinically relevant as a prognostic marker in the treatment of SCLC patients.

## Supplementary Information

**Additional file 1: Supplementary Figure 1**. Funnel plot for the analysis of publication bias in studies evaluating neutrophil-to-lymphocyte ratio (NLR) as prognostic markers of overall survival in patients with small cell lung cancer. HR, hazard ratio.

**Additional file 2: Supplementary Figure 2**. Funnel plot for the analysis of publication bias in studies evaluating platelet-to-lymphocyte ratio (PLR) as prognostic markers of overall survival in patients with small cell lung cancer. HR, hazard ratio.

## Data Availability

The datasets used and/or analysed during the current study are available from the corresponding author on reasonable request.

## Abbreviations

ALI: Advanced lung cancer inflammation index
CI: Confidence interval
CRP: c-reactive protein
ED: Extended disease
HALP: Haemoglobin, albumin, lymphocyte, and platelet score
HR: Hazard ratio
LD: Limited disease
LMR: Lymphocyte-to-monocyte ratio
mGPS: Modified Glasgow prognostic score
MLR: Monocyte-to-lymphocyte ratio
NLR: Neutrophil-to-lymphocyte ratio
NSCLC: Non-small lung cancer
OS: Overall survival
PLR: Platelet-to-lymphocyte ratio
PRISMA: Preferred Reporting Items for Systematic Reviews and Meta-Analyses
QUIPS: Quality of Prognosis Studies Tool
SII: Systemic immune-inflammation index SCLC Small cell lung cancer
