# Supplementary figures and images for: Inflammation scores as prognostic biomarkers in small cell lung cancer: a systematic review and meta-analysis
Source: Syst Rev. 2021 Jan 28;10:40. doi: 10.1186/s13643-021-01585-w (PMC7844954; doi:10.1186/s13643-021-01585-w)

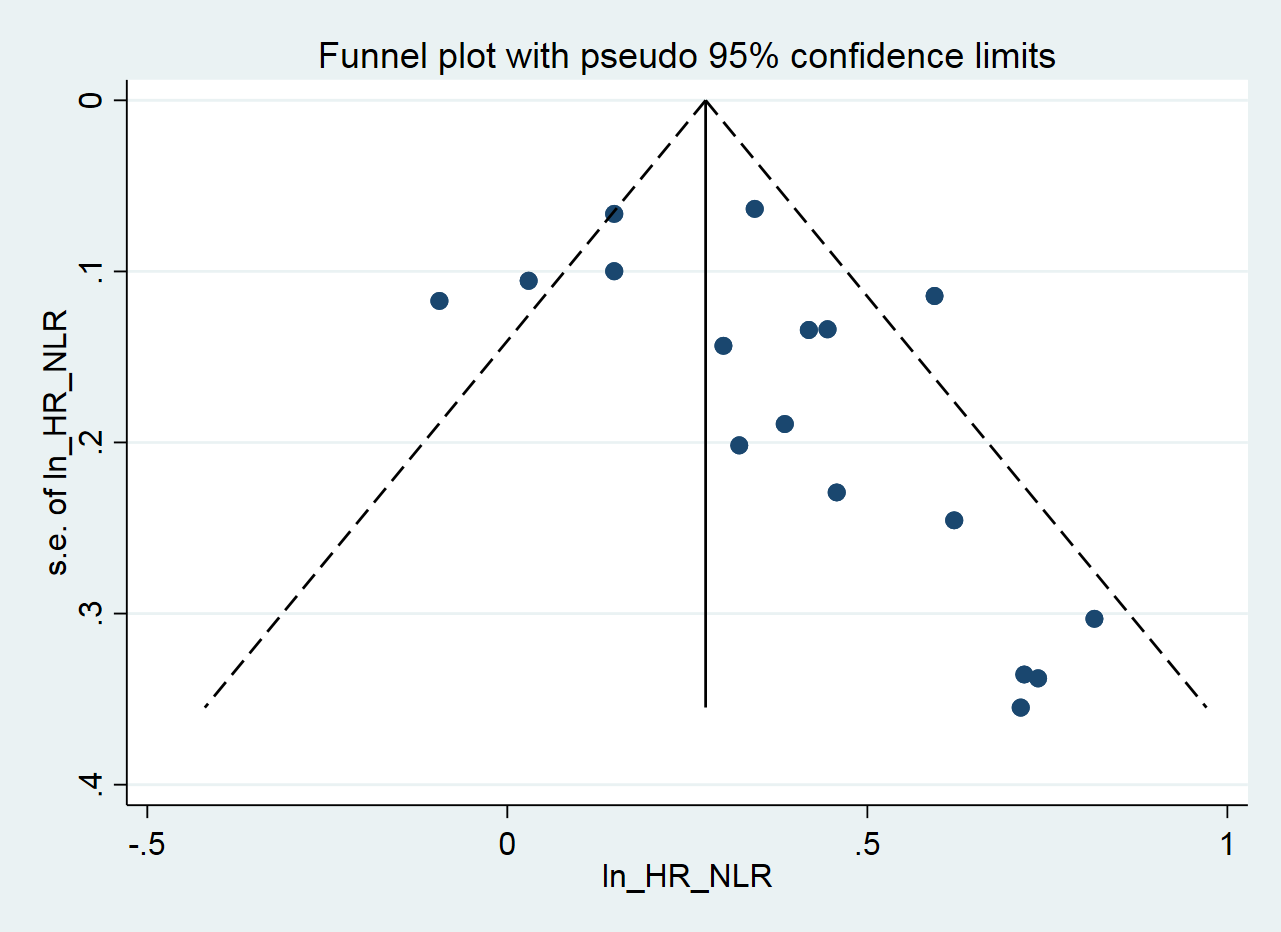

Supplement: Supplementary file 1 — Additional file 1: Supplementary Figure 1. Funnel plot for the analysis of publication bias in studies evaluating neutrophil-to-lymphocyte ratio (NLR) as prognostic markers of overall survival in patients with small cell lung cancer. HR, hazard ratio. [file 13643_2021_1585_MOESM1_ESM.tif]

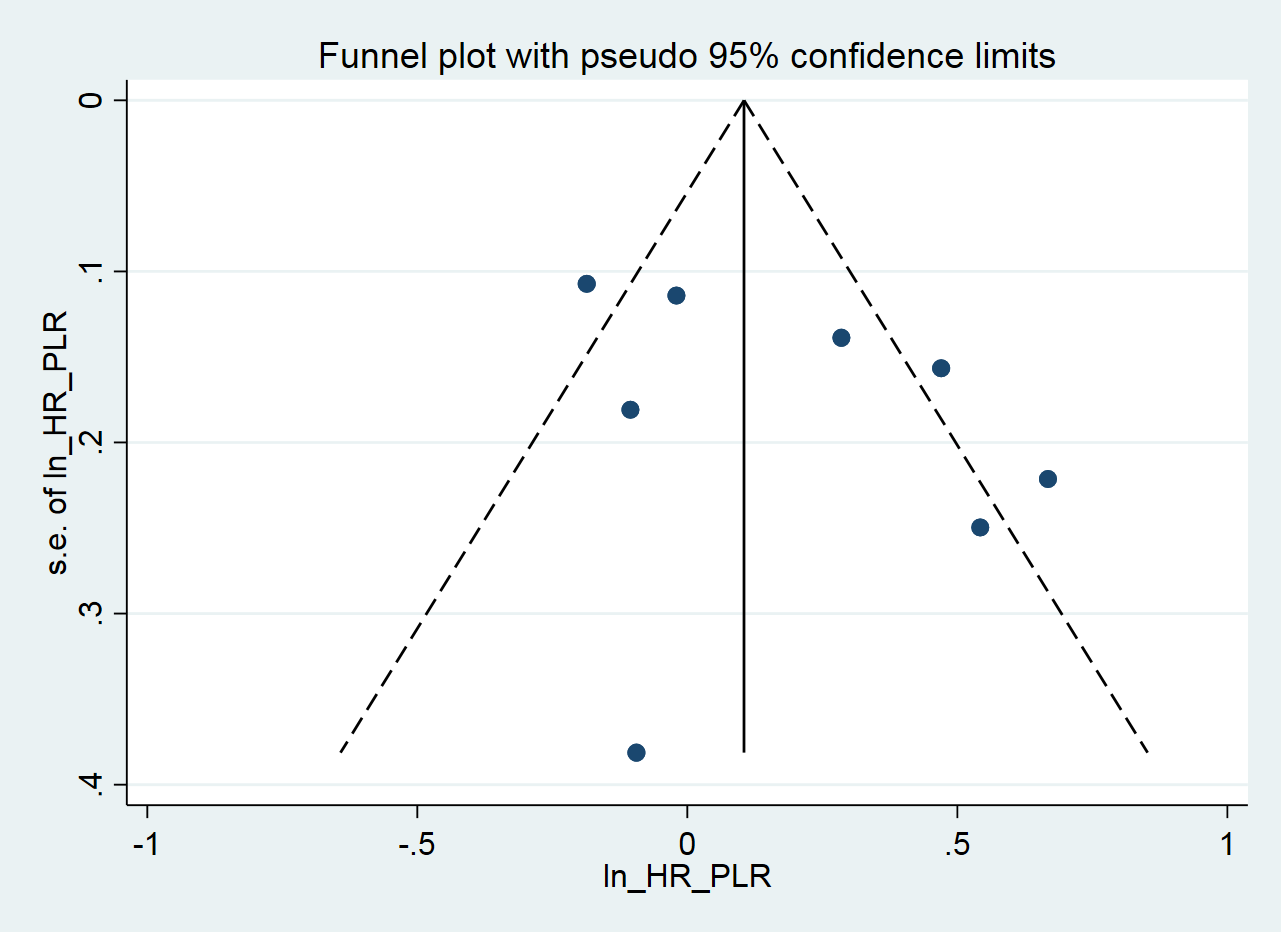

Supplement: Supplementary file 2 — Additional file 2: Supplementary Figure 2. Funnel plot for the analysis of publication bias in studies evaluating platelet-to-lymphocyte ratio (PLR) as prognostic markers of overall survival in patients with small cell lung cancer. HR, hazard ratio. [file 13643_2021_1585_MOESM2_ESM.tif]
